# Development and Validation of a Risk Model for Prediction of Hazardous Alcohol Consumption in General Practice Attendees: The PredictAL Study

**DOI:** 10.1371/journal.pone.0022175

**Published:** 2011-08-10

**Authors:** Michael King, Louise Marston, Igor Švab, Heidi-Ingrid Maaroos, Mirjam I. Geerlings, Miguel Xavier, Vicente Benjamin, Francisco Torres-Gonzalez, Juan Angel Bellon-Saameno, Danica Rotar, Anu Aluoja, Sandra Saldivia, Bernardo Correa, Irwin Nazareth

**Affiliations:** 1 Department of Mental Health Sciences, University College London, London, United Kingdom; 2 Medical Research Council General Practice Research Framework, London, United Kingdom; 3 Department of Primary Care and Population Health, University College London, London, United Kingdom; 4 Department of Psychiatry, University of Granada, Granada, Spain; 5 El Palo Health Centre, Department of Preventive Medicine, Malaga, Spain; 6 Department of Family Medicine, University of Ljubljana, Ljubljana, Slovenia; 7 Faculty of Medicine, University of Tartu, Tartu, Estonia; 8 University Medical Center, Utrecht, The Netherlands; 9 Centro de Estudos de Doencas Cronicas, Faculdade de Ciências Médicas, Universidade Nova de Lisboa, Lisbon, Portugal; 10 Departamento de Psiquiatría y Salud Mental, Universidad de Concepción, Concepción, Chile; Fred Hutchinson Cancer Research Center, United States of America

## Abstract

**Background:**

Little is known about the risk of progression to hazardous alcohol use in people currently drinking at safe limits. We aimed to develop a prediction model (predictAL) for the development of hazardous drinking in safe drinkers.

**Methods:**

A prospective cohort study of adult general practice attendees in six European countries and Chile followed up over 6 months. We recruited 10,045 attendees between April 2003 to February 2005. 6193 European and 2462 Chilean attendees recorded AUDIT scores below 8 in men and 5 in women at recruitment and were used in modelling risk. 38 risk factors were measured to construct a risk model for the development of hazardous drinking using stepwise logistic regression. The model was corrected for over fitting and tested in an external population. The main outcome was hazardous drinking defined by an AUDIT score ≥8 in men and ≥5 in women.

**Results:**

69.0% of attendees were recruited, of whom 89.5% participated again after six months. The risk factors in the final predictAL model were sex, age, country, baseline AUDIT score, panic syndrome and lifetime alcohol problem. The predictAL model's average c-index across all six European countries was 0.839 (95% CI 0.805, 0.873). The Hedge's g effect size for the difference in log odds of predicted probability between safe drinkers in Europe who subsequently developed hazardous alcohol use and those who did not was 1.38 (95% CI 1.25, 1.51). External validation of the algorithm in Chilean safe drinkers resulted in a c-index of 0.781 (95% CI 0.717, 0.846) and Hedge's g of 0.68 (95% CI 0.57, 0.78).

**Conclusions:**

The predictAL risk model for development of hazardous consumption in safe drinkers compares favourably with risk algorithms for disorders in other medical settings and can be a useful first step in prevention of alcohol misuse.

## Introduction

Hazardous drinking, defined as alcohol consumption that places a person at risk of adverse health events, is a leading contributor to the global burden of disease [1]. Prevalence in some populations is as high as 29 per cent [2]. Hazardous drinking was defined in terms of excessive consumption [21 drinks or more per week for men (or ≥7 drinks per occasion at least 3 times a week), and 14 drinks or more per week for women (or ≥5 drinks per occasion at least 3 times a week)] [2] or in terms of a score of 8 or over on the Alcohol Use Disorders Identification Test (AUDIT) [3]. More recent validation studies of the AUDIT, however, have recommended tailored cut off points according to gender. The suggested optimal cut off of AUDIT scores is ≥8 in males and ≥5 in women [4].

Although we know a great deal about detection [5]–[7] and approaches to treatment [8], [9] of hazardous or dependent drinking, we know much less about risk of progressing to hazardous use in people currently drinking at safe limits. In particular, although many risk factors are well recognised [10]–[13], effective prevention is hindered by lack of evidence about their combined effect. Our objectives were to develop a risk model (predictAL) for the future development of hazardous drinking in safe drinkers attending European general practices and test its predictive power in a non-European setting. We took the approach of risk models developed to predict onset of cardiovascular disease [14] and risk of major depression (predictD) [15], both of which provide a percentage risk estimate over a given time period.

## Methods

### Study setting and design

To develop the predictAL model we used data from a prospective cohort of general practice attenders which had been established to develop a risk model (predictD) for the development of major depression[15], [16]. The research was approved in the lead centre (UK) by the South East Multi-centre Research Ethics Committee and by key ethical committees in each of the other centres. The study was conducted in six European countries: 1) 25 general practices in the Medical Research Council's General Practice Research Framework, in the United Kingdom; 2) nine large primary care centres in Andalucía, Spain; 3) 74 general practices nationwide in Slovenia; 4) 23 general practices nationwide in Estonia; 5) seven large general practice centres near Utrecht, The Netherlands; and 6) two large primary care centres in the Lisbon area of Portugal. We assessed the external validity of the risk model in patients attending 78 doctors in 10 general practice centres in Concepción and Talcahuano in the Eighth region of Chile. General practices covered urban and rural populations with considerable socio-economic variation.

### Study participants

General practice attenders aged 18 to 75 were recruited in Europe between April 2003 and September 2004 and in Chile between October 2003 and February 2005. Exclusion criteria were an inability to understand one of the main languages involved, psychosis, dementia and incapacitating physical illness. Recruitment differed slightly in each country because of local service preferences. In the UK and the Netherlands, researchers spoke to patients directly while they waited to see practice staff. In remaining European countries doctors introduced the study to patients before they saw the researchers. In Chile attenders were stratified on age and gender according to figures for the populations served by each health centre and participants selected randomly within each stratum. Participants gave informed consent and undertook a research evaluation within two weeks. All assessments at baseline and both follow-up points were conducted by face-face interview at the practices or in respondents' homes.

### Measurement of hazardous drinking and associated risk factors

Alcohol use in the preceding six months was assessed using the AUDIT [17], a tool for detection of alcohol use disorders in general practice [7]. It is a widely used and well validated instrument that contains 10 questions about use of, and attitudes to, alcohol consumption over the preceding six months. We defined hazardous drinking on AUDIT scores of 8 or more in men and 5 or more in women [4].

Few studies have attempted to measure key risk factors for the development of hazardous drinking in abstinent or safe drinkers. In our establishment of the predict cohort, we measured a wide range of risk factors that were known to be associated with the onset of major depression [15]. The fact that many of these medical, psychological and social factors are also known to be associated with alcohol misuse in the literature [10], [11], [11], [18,19], also made it possible for us to model risk of hazardous drinking. Where possible, in the predict study we used standardised measures. Questions taken or adapted from published questionnaires or developed for the study were evaluated for test-retest reliability in 285 general practice attendees recruited equally across the European countries before the main study began [16]. Each instrument or question not available in the relevant languages was translated from English and back-translated by professional translators [16]. The 38 candidate risk factors are listed numerically as RF1–38. Those subjected to test-retest reliability are shown in italics; agreement was high [16].

- Age (RF1), sex (RF2), occupation (RF3), educational level (RF4), marital status (RF5), employment status (RF6), ethnicity (RF7), living alone or with others (RF8), born in country of residence or abroad (RF9) and long standing physical illness (RF10).
- A DSMIV diagnosis of major depression in the preceding six months was made using the Depression Section of the Composite International Diagnostic Interview (CIDI) (RF11) [20], [21].
- Life-time depression was based on affirmative answers to both of the first two questions of the CIDI depression section (RF12).
- *Stress in paid and unpaid work in the preceding six months using questions from the job content instrument* [*22*] *. Participants were categorised as feeling in control in paid work (RF13) or unpaid work (RF14); as experiencing difficulties without support in paid or unpaid work (RF15); and experiencing distress without feeling respect for their paid or unpaid work (RF16).*
- Financial strain using a question used in UK government social surveys(RF17) [23].
- Besides the 10 AUDIT questions we asked whether participants had ever had problems with drinking too much alcohol or had ever received treatment for an alcohol problem (RF18).
- AUDIT score at baseline (RF19). Binge drinking at baseline was taking from responses to question three of the AUDIT. Binge drinking was defined as “having six or more drinks on one occasion” at least monthly (RF20).
- Self-rated physical (RF21) and mental health (RF22) were assessed by the Short Form 12 [24]. The weights used to calculate scores are from version 1.
- *Whether participants had ever used recreational drugs using adapted sections of the CIDI (RF23).*
- We asked whether participants currently smoked cigarettes, cigars or a pipe (RF24). It was not possible to collect smoking data in the Netherlands and Estonia (see statistical analysis below).
- *Questions on the quality of sexual (RF25) and emotional relationships(RF26) with partners or spouses* [*25*] *.*
- *Presence of serious physical, psychological or substance misuse problems, or any serious disability, in people who were in close relationship to participants (RF27).*
- *Difficulties in getting on with people and maintaining close relationships (RF28)* [*26*] *.*
- Childhood experiences of physical and/or emotional (RF29) and sexual abuse (RF30) [27].
- Holding religious and/or spiritual beliefs (RF31) [28].
- *History of serious psychological problems (RF32) or suicide in first-degree relatives (RF33)* [*29*].
- Anxiety (RF34) and panic symptoms (RF35) in the previous six months using relevant sections of the Patient Health Questionnaire (PHQ) [30].
- Major life events in the preceding six months (RF36), using the List of Threatening Life Experiences Questionnaire [31].
- *Experiences of discrimination (RF37) in the preceding six months on grounds of sex, age, ethnicity, appearance, disability or sexual orientation using questions from a European study* [*32*].
- Adequacy of social support (RF38) from family and friends [33].

### Main outcome

All participants were re-evaluated after six months using the AUDIT.

### Statistical analysis

#### Data imputation

Missing data in all variables were imputed using the method of chained equations, implemented in the Stata command ice [34]. This involves using regression models to determine plausible values for the missing data, starting with variables that had the lowest percentage of missing data and continuing until all variables are imputed. Continuous variables were imputed using multiple linear regression. Dichotomous variables were imputed using logistic regression and nominal variables such as employment status, education status, control in paid work, discrimination, problems with someone close, satisfaction with emotional relationship with spouse or partner were imputed using multinomial logistic regression. Number of life events was imputed using ordered logistic regression. This process was carried out ten times (cycles) resulting in one imputed dataset. Then the whole process was repeated to give ten imputed datasets to allow variability due to uncertainty of the exact values [35]. The final imputation model consisted of all variables listed above (with the exception of smoking status, the reasons for which are explained later) as well as the outcome included as a continuous score and then dichotomised before analysis. Each imputed dataset was analysed separately and estimates were combined using Rubin's rules [36].

#### Preliminary steps

Before building the multivariable model we undertook two preliminary steps. 1) Data on smoking history was collected as an additional part of the original PREDICT study. The cost incurred for this aspect of the data collection was covered by funds obtained independently by each participating centre but this was not possible in the Netherlands and Estonia. We hence first analysed data from the four European countries that were able to collect a smoking history as we believed this was very likely to be an important predictor variable. However, when current smoking (risk factor 24 above) showed no association with development of hazardous drinking we dropped this risk factor from the analysis. 2) A rule of thumb for estimating sample sizes for developing prognostic models is that there should be at least 10 events for each variable entered in the model [37]. Thus, given the event rate of hazardous drinking, we did not enter all 38 predictor variables into the model. Instead, we first conducted a series of univariable analyses to select out those variables that were not significant at the p<0.1 level. The remaining variables were then entered into the full multivariable model. These were AUDIT score at baseline; age at baseline; SF12 physical health score; SF12 mental health score; sex; professional status; educational status; marital status; employment status; living alone; lifetime alcohol problem; ever used recreational drugs; satisfaction with sex life; satisfaction with emotional relationship with spouse/partner; physical or emotional child abuse; religious/spiritual beliefs; presence of panic syndrome; binge drinking and country of residence of each participant.

#### Model building

We developed our multivariable predictAL model in the imputed data for safe or abstinent drinkers (male AUDIT score ≤8 and females AUDIT score <5) by examining the 19 remaining predictor variables at baseline in a stepwise logistic regression with robust standard errors to adjust for general practice clustering. We used a conservative threshold for inclusion of p<0.01 in order to produce a stable model and minimise the degree of over-fitting. We retained age and sex in all regression models because of their well known associations with development of hazardous drinking [18]. We also retained country because of an *a priori* assumption of clustering within country. Multivariable fractional polynomial analysis was used to assess possible non-linear effects of continuous predictors. Pair wise interactions between the variables in the model and sex were tested. The resulting predictAL score provides a predicted probability of hazardous alcohol consumption developing over six months.

#### Internal validation

We calculated the c-index [38] to estimate the discriminative power of the final predictAL model in each European country and all European countries combined. We adjusted for over-fitting of our model by computing a shrinkage factor based on the initial model including all 19 variables and applied it to the model coefficients [39]. We assessed the goodness of fit of the final predictAL model by grouping individuals into deciles of predicted risk and comparing the observed probability of hazardous drinking within these groups with the average risk. We calculated effect sizes using Hedge's g [40] for the difference in log odds of predicted probability between patients who were later observed to be hazardous drinkers and those who were not. Finally we report the threshold values of risk score, and the associated sensitivity, for a range of specificity that would be practical (minimising false positives) when using the instrument in a clinical setting. We stress that these values are for the fitted European model (not the external population) so we might expect them to be worse in practice.

#### External validation

We used the c-index, Hedge's g and a comparison of predicted versus observed probability of hazardous drinking, to evaluate the performance of the predictAL model in the Chilean data.

All analyses and data imputation were performed using Stata release 11 [41].

## Results

### Response rates and missing data

**Figure 1:**
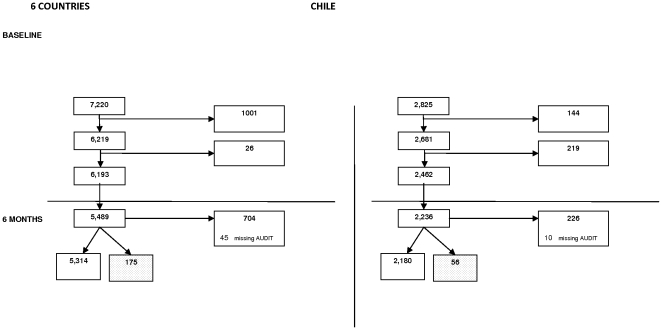
Flow chart of patients through the study.

15, 205 people attending their general practitioners were approached of whom 10,045 people (69%) took part in the seven countries [16]. Response to recruitment was high in Portugal (76%), Spain (87%), Estonia (80%), Slovenia (80%) and Chile (97%) but lower in the UK (44%) and the Netherlands (45%). Ethical considerations prevented the collection of data on non-responders at baseline. Across all countries the response to the six months follow-up was 89.5%. 6193 European and 2462 Chilean attenders recorded AUDIT scores below 8 in men or below 5 in women at recruitment and thus were involved in the modelling of risk (Figure 1, table 1).

**Table 1:** Demographic characteristics of the study population.

Variable	Europe 6		UK		Spain		Slovenia		Estonia		Netherlands		Portugal		Chile	
N (% of European sample)	6193	100	1016	16	1170	19	1035	17	898	15	967	16	1107	18	2462	
	N	%	n	%	n	%	N	%	n	%	n	%	n	%	n	%
**Hazardous drinking at 6 months**																
Yes	175	3	56	6	13	1	14	1	24	3	58	6	10	1	56	2
No	5314	86	829	82	917	78	934	90	829	92	822	85	983	89	2180	89
Missing	704	11	131	13	240	21	87	8	45	5	87	9	114	10	226	9
**Sex**																
Male	1941	31	360	35	331	28	354	34	206	23	357	37	333	30	590	24
Female	4252	69	656	65	839	72	681	66	692	77	610	63	774	70	1872	76
**Age** mean (SD)	49	(15)	54	(14)	50	(15)	49	(14)	43	(16)	49	(15)	50	(15)	47	(15)
**Professional status**																
Professional	4860	78	732	72	1057	90	876	85	601	67	591	61	1003	91	2426	99
Not professional	1202	19	256	25	112	10	156	15	251	28	323	33	104	9	32	1
Missing	131	2	28	3	1	0.1	3	0.3	46	5	53	5	0	0	4	0.2
**Education**																
Higher	1739	28	383	38	144	12	167	16	519	58	385	40	141	13	81	3
Secondary	2007	32	422	42	240	21	386	37	276	31	479	50	204	18	929	38
Primary or no education	1955	32	26	3	782	67	237	23	103	11	79	8	726	66	1140	46
Trade or other	448	7	165	16	2	0.2	245	24	0	0	0	0	36	3	310	13
Missing	44	1	20	2	0	0	0	0	0	0	24	2	0	0	2	0.1
**Marital status**																
Married or living together	4471	72	774	76	822	70	739	71	613	68	718	74	805	73	1418	58
Not married or living together	1703	28	241	24	347	30	293	28	285	32	236	24	301	27	1044	42
Missing	19	0.3	1	0.1	1	0.1	3	0.3	0	0	13	1	1	0.1	0	0
**Employment**																
Employed or full time student	3083	50	480	47	386	33	550	53	640	71	502	52	525	47	837	34
Retired	1463	24	305	30	188	16	381	37	142	16	139	14	308	28	197	8
Other	1611	26	231	23	595	51	97	9	116	13	299	31	273	25	1428	58
Missing	36	1	0	0	1	0.1	7	1	0	0	27	3	1	0.1	0	0
**Ethnicity**																
White European	5996	97	945	93	1157	99	1030	100	897	100	875	90	1092	99	0	0
Not white European	131	2	33	3	11	1	3	0.3	1	0.1	68	7	15	1	2462	100
Missing	66	1	38	4	2	0.2	2	0.2	0	0	24	2	0	0	0	0
**Household status**																
Living alone	691	11	137	13	74	6	124	12	96	11	171	18	89	8	104	4
Not living alone	5502	89	879	87	1096	94	911	88	806	89	796	82	1018	92	2358	96
**Born in country of residence**																
Yes	5654	91	940	93	1119	96	817	79	824	92	880	91	1074	97	2451	100
No	459	7	72	7	48	4	214	21	30	3	62	6	33	3	7	0.3
Missing	80	1	4	0.4	3	0.3	4	0.4	44	5	25	3	0	0	4	0.2

### Numbers in the modelling

Once current smoking was eliminated as a significant predictor of risk in the four European countries that collected those data (see analysis section), the predictAL algorithm was developed using data for the 6193 attenders in all six European countries that had AUDIT scores below 8 in men and 5 in women at recruitment. Validation was carried out using six-month outcome data on 2462 exactly similar attenders in Chile (figure 1, table 1). The amount of missing data in outcome and covariates is summarised in table 2. For all countries there were few outcome data missing at baseline, but this increased to 11% after six months in Europe. Taking the set of covariates as a whole, a large proportion of individuals were missing data in at least one covariate. 56% of participants in the six European countries and 67% in Chile were missing data in at least one covariate. However, restricting the set of covariates to only those used in the final model, this proportion decreases to 1% and 0.1%.

**Table 2:** Missing data in outcome and covariates in Europe and Chile.

	Europe6 (N = 6193)		Chile (N = 2462)	
	n	%	N	%
Missing hazardous drinking six months				
No	5489	89	2236	89
Yes	704	11	226	9
Missing data from any covariate				
No	5063	82	2400	97
Yes	1130	11	62	3
Missing data from any covariate in the final model				
No	6140	99	2460	100
Yes	53	1	2	0.1

### Onset of hazardous drinking

We estimated that the incidence of hazardous drinking over six months in Europe was 4.0% (95% CI: 3.4%, 4.5%) and in Chile was 2.7% (CI 2.0%, 3.3%). The figures given here vary very slightly from table 1 as they are based on imputed data.

### Development of the predictAL algorithm in Europe

Three variables (baseline AUDIT score, panic syndrome and lifetime alcohol problem) in addition to sex, age and country were retained at p<0.01 after the backwards elimination procedure (table 3). No interactions between sex and other variables in the model were significant. AUDIT score and lifetime alcohol problem was found in each of the 10 imputed data sets. The additional variables to appear were panic syndrome in six imputed datasets, marital status in four and, having ever used recreational drugs in one. Thus, the model was stable in terms of the variables selected.

**Table 3:** PredictAL model derived in the imputed European datasets.

Variable	Coefficient	SE	Coefficient after shrinkage*	p-value
Constant	−4.783	0.540	−4.411	<0.001
AUDIT score at baseline	0.722	0.066	0.640	<0.001
Age (years)	−0.021	0.005	−0.019	<0.001
Female sex	1.503	0.268	1.344	<0.001
Lifetime alcohol problem	0.880	0.242	0.787	<0.001
Panic	0.669	0.254	0.598	0.008
Country				
United Kingdom	Reference			
Spain	−0.823	0.293	−0.736	0.006
Slovenia	−0.983	0.277	−0.879	<0.001
Estonia	−1.082	0.274	−0.968	<0.001
Netherlands	−0.158	0.202	−0.141	0.437
Portugal	−1.212	0.597	1.084	0.043
6 country average	−0.710			
Chile			−0.344	

*Shrinkage factor 0.894.

The c-index for the predict Al model in all the European countries was 0.839 (95% CI 0.0805 to 0.873) (Table 4). The effect size for the difference in log odds of predicted probability between attenders in Europe who subsequently developed hazardous alcohol use and those who did not was 1.38 (95% CI 1.25, 1.51) (table 5). The model discriminated best in the UK, the Netherlands and Spain and least well in Slovenia and Portugal.

**Table 4:** C-index statistics for the predictAL model each country#.

Country	c-index (95% confidence intervals)
All European	0.839 (0.805, 0.873)
UK	0.807 (0.764, 0.850)
Spain	0.793 (0.718, 0.867)
Slovenia	0.764 (0.696, 0.831)
Estonia	0.817 (0.765, 0.870)
Netherlands	0.830 (0.788, 0.871)
Portugal	0.759 (0.647, 0.871)
Chile*	0.781 (0.717, 0.846)

#The c-index is also known as the Area under the Relative operating Characteristic (ROC) Curve of sensitivity against 1- specificity. A perfect test has a c-index of 1.00 while a test which performs no better than chance has a c-index of 0.5.

•Risk score computer using unshrunk estimates in Europe and shrunk estimates in Chile.

**Table 5:** Effect sizes computed using Hedge's g#.

Country	Effect size (95% confidence intervals)
Europe6	1.38 (1.25, 1.51)
UK	1.24 (1.04, 1.43)
Spain	1.35 (0.86, 1.85)
Slovenia	0.99 (0.51, 1.46)
Estonia	1.16 (0.81, 1.52)
Netherlands	1.29 (1.13, 1.44)
Portugal	0.91 (0.26, 1.56)
Chile	0.68 (0.57, 0.78)

#Predicted probabilities were logarithmically transformed and compared between participants who developed hazardous drinking and those who did not over the subsequent six months. Hedge's g is preferred to Cohen's d where the sizes of each group are arkedly unequal.

**Figure 2:**
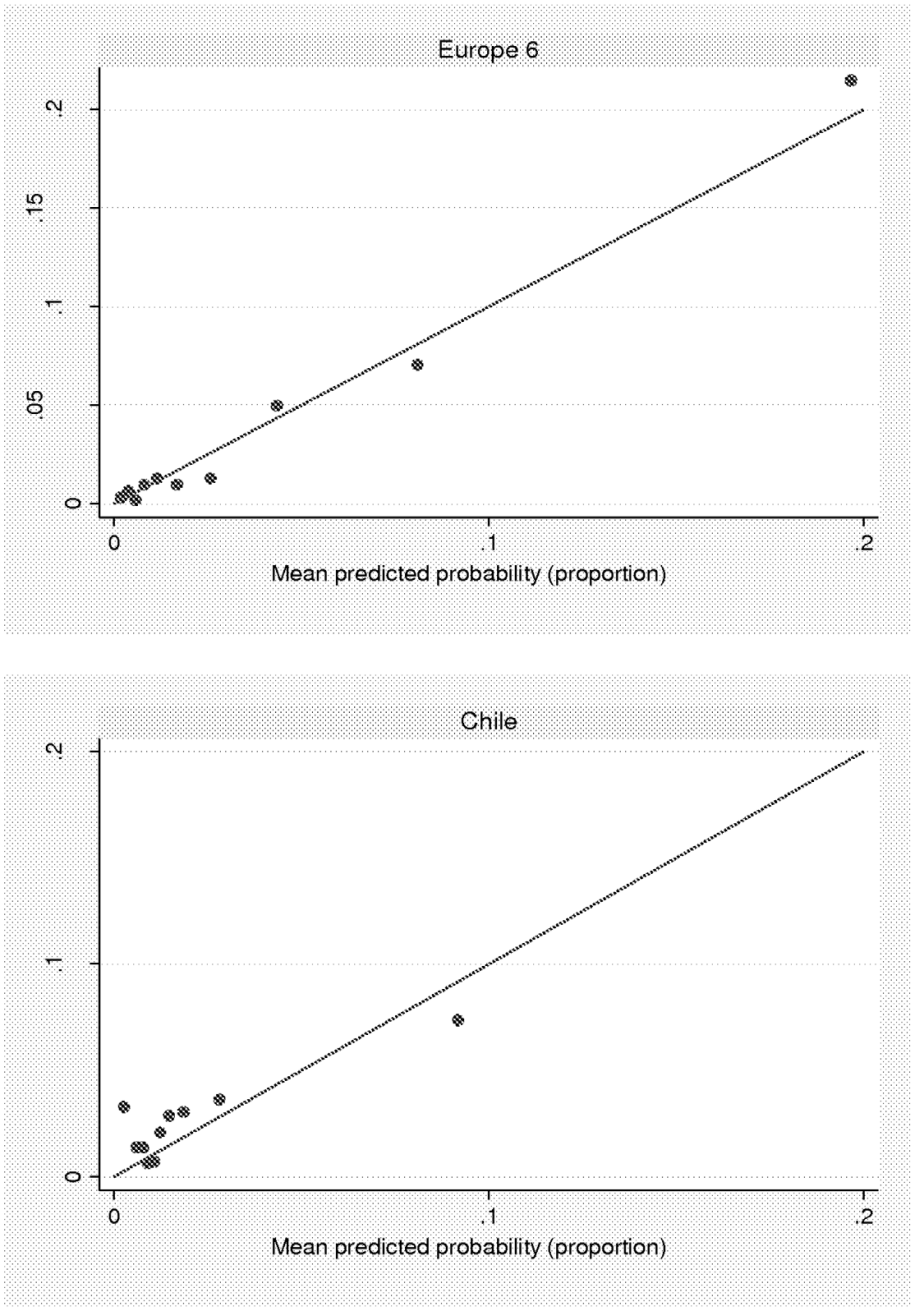
Mean predictAL score plotted against observed probability of hazardous drinking (within deciles of the predictAL score).

To examine the fit of the predictAL model, we divided the European population into deciles of predicted probability of hazardous drinking. Within each decile we plotted mean risk score at recruitment against observed probability of hazardous drinking at six months (figure 2), using the model coefficients shown in table 3. The plot for Europe shows that onset of hazardous drinking in the highest decile of risk score in Europe was approximately 21% in contrast to the overall incidence of 4%.

Estimates of sensitivity and specificity of the predictAL score in predicting the development of hazardous drinking over 6 months are shown in table 6. Examples of participants screening at increasing levels of predicted probability of hazardous alcohol use on the predictAL algorithm are shown in Box S1.

**Table 6:** Thresholds for specificity and sensitivity in each setting.

	Predicted probability of hazardous drinking (predictAL risk score)	specificity	sensitivity
Europe	0.061	0.800	0.749
Europe	0.079	0.850	0.665
Europe	0.0107	0.900	0.594
*Country*			
UK	0.121	0.800	0.722
UK	0.151	0.850	0.542
UK	0.179	0.900	0.431
Spain	0.027	0.800	0.600
Spain	0.034	0.850	0.600
Spain	0.048	0.900	0.520
Slovenia	0.036	0.800	0.727
Slovenia	0.044	0.850	0.636
Slovenia	0.061	0.900	0.500
Estonia	0.060	0.800	0.613
Estonia	0.068	0.850	0.581
Estonia	0.091	0.900	0.516
Netherlands	0.127	0.800	0.771
Netherlands	0.144	0.850	0.714
Netherlands	0.170	0.900	0.557
Portugal	0.022	0.800	0.474
Portugal	0.029	0.850	0.474
Portugal	0.045	0.900	0.421
Chile	0.022	0.800	0.415
Chile	0.027	0.850	0.403
Chile	0.038	0.900	0.279

### External validation of the predictAL algorithm in Chile

The predictAL model was validated in Chile using data provided by the 2462 attenders who were abstinent or safe drinkers at recruitment. In Chile 2% of such people reported hazardous drinking by the 6 months follow-up point. Predicted risks at six months for Chile were obtained using shrunk coefficients. Because country is included in the model, it was necessary to recalibrate the model in Chile. In Chile the c-index for the predictAL model was 0.781 (95% CI 0.717, 0.846) and Hedge's g was 0.68 (95% CI 0.57, 0.78) (tables 4 and 5).

### Sensitivity analysis

The inclusion of country as a variable in the predictAL model accounts for variation between countries in the risk assessment. However, given the relatively lower recruitment rates in the UK and the Netherlands, and their somewhat higher incidence rates of hazardous drinking at 6 months (Table 1), we conducted a sensitivity analysis to see whether exclusion of participants from the UK and the Netherlands changed our prediction model. There were minimal changes in the coefficients for most variables in the model with the exception of country which was no longer significant (data available from the authors on request).

## Discussion

PredictAL is a brief risk assessment for the development of hazardous drinking over six months, which was developed in general practice in Europe and validated in attenders in Chile. We emphasise that we were not attempting to provide a superior instrument for detection of *current* hazardous drinking; rather we have developed an algorithm to estimate *future* risk of hazardous drinking. It is accurate with c-indices equal to or above those usually reported for risk prediction in medicine, such as cardiovascular events [42]. The risk factors involved (sex, age, country, baseline AUDIT score, lifetime alcohol problem and the presence of panic syndrome) are not surprising. Our study was not a search for new risk factors; rather it was an attempt to gauge how they might most parsimoniously be combined to model risk in medical settings. The absence of what might safely be regarded as key risks, such as cigarette smoking, is also not surprising. Modelling risk in this way gives prominence to those risk factors that trump others. When the algorithm is applied in a country besides the six in Europe, or Chile, we recommend using either the overall European coefficient (−0.710) or the coefficient for the country that most closely matches the six months incidence of hazardous drinking (if known) in the new setting (table 3). The coefficient for Chile was obtained by a recalibration of the predictAL model in that country.

### Strengths and limitations

The main strength of our study is that we have developed the predictAL model in one continent and rigorously validated it in another. The c-index provides a standardised way of comparing the discriminative power of tests that use different measurement units in different settings [43] and shows that predictAL compares very favourably with risk instruments for other health problems. However, our study has a number of limitations. Lower recruitment rates in the UK and the Netherlands possibly occurred because the study was not so obviously introduced by the doctors. Nevertheless, response to follow-up in all countries was high and our sensitivity analysis excluding participants from these countries suggests responders were not a particular or unusual group. One strength of using data from a cohort that was established originally to develop a risk model for major depression [15], is that participants were unaware of the aim behind this risk modelling. Including the baseline AUDIT score as a covariate in the model takes account of the dependence between baseline and six month data. Although it might be argued that six months is a relatively short time over which to estimate risk, we believe that it is a pragmatic choice in general practice where longer term prediction may be less salient to patients and doctors. Using a two step process in which variables not likely to enter the model were first removed, reduced the impact of the low event rate of hazardous drinking on the power of our analysis. Finally, although a 3% incidence of hazardous drinking is low from the statistical point of view, this degree of conversion from normal to hazardous drinking over only six months presents a significant clinical risk. Until now we have had no tools whatsoever to predict normal drinkers who are at risk of future hazardous use and our efforts at prevention are also rudimentary. Hence, we believe our analysis adds valuable information to a field in need of innovation.

### Application in clinical practice

Efforts to deal with the public health and social consequences of hazardous drinking must include a focus of prevention. The questions in predictAL are brief and risk scores can readily be calculated using the algorithm (appendix). Panic disorder is often established before the age of 20 and thus is an early predictor of alcohol misuse that is open to intervention [44]. Furthermore our work shows the potential for extending the AUDIT beyond its usual function of detecting current hazardous and dependent drinkers into the realm of predicting risk of hazardous drinking in so-called safe drinkers. Our results expressed by the c-indices and effect sizes demonstrate a clear difference in risk between safe drinkers who became hazardous drinkers six months later and those who did not. Thus when family doctors use the AUDIT to screen for hazardous alcohol use in their patients they might also consider adding in two extra pieces of information. The first is whether their patient has ever had problems drinking too much alcohol or has ever received treatment for an alcohol problem, and the second is a brief review of panic symptoms experienced in the previous six months (derived from Patient Health Questionnaire 30) [30]. This additional information will enable primary care clinicians to assess the *future* risk of hazardous drinking in men with AUDIT scores of 8 or less and in women with scores of 5 or less.

In reporting a range of thresholds for sensitivity and specificity (table 6) we would recommend maximising specificity at the cost of reduced sensitivity to minimise the potential workload for family doctors engaging with false positives. For example, if primary care physicians were to use a European threshold for risk of 10.7% (i.e. specificity of 0.9 and sensitivity of 0.594) they could be sure that the numbers of patients falsely identified as at risk of hazardous drinking (false positives) will kept to a minimum. Although this would be at the cost of missing some of those who would go on to develop hazardous drinking over six months, use of a high cut off ensures that prevention efforts are less likely to be wasted on those not at risk of becoming hazardous drinkers. However, if prevention interventions require little input by way of physician time and effort (e.g. a web-based alcohol self-help prevention package), a lower cut off of 6.1% might be considered, as the larger number of positives caught in the net could be offered the intervention without substantially increasing costs to the health service.

We acknowledge that many general practitioners have difficulty dealing with current hazardous use but this difficulty does not detract from efforts to predict hazardous use in advance. In fact, successful prediction may reduce the more challenging work that general practitioners are frequently called on to do with people already drinking unsafely. Patients identified as at risk on screening could be flagged on practice computers to alert practice staff when they attend. Recognition of those at risk may be helpful when it leads to watchful waiting or active support with advice on social and behavioural strategies they might use to reduce their risk. There is controlled trial evidence that shows providing information on coping with anxiety and the consequences of hazardous drinking may prevent alcohol misuse in young people [45]. The application of strategies for the prevention of hazardous drinking in primary care would benefit from further study.

### Conclusions

This predictAL risk model for development of hazardous consumption in safe drinkers compares favourably with risk algorithms used in other medical settings and may be useful in prevention of alcohol disorders. We also suggest that this is an advance that takes the AUDIT beyond simply the detection of current hazardous use.

## Supporting Information

Box S1 **Examples of a range of predicted probabilities of hazardous drinking at baseline.** AUDIT scores of 8 or more in men and 5 or more in women were defined as hazardous drinking.
(DOCX)
