# Supplementary material for: Development and Validation of a Risk Model for Prediction of Hazardous Alcohol Consumption in General Practice Attendees: The PredictAL Study
Source: PLoS One. 2011 Aug 10;6(8):e22175. doi: 10.1371/journal.pone.0022175 (PMC3154188; doi:10.1371/journal.pone.0022175)
Supplement: Box S1 — Examples of a range of predicted probabilities of hazardous drinking at baseline. AUDIT scores of 8 or more in men and 5 or more in women were defined as hazardous drinking. (DOCX) [file pone.0022175.s001.docx]

**Box Examples of a range of predicted probabilities of hazardous drinking at baseline**

**Risk score (predicted probability of hazardous drinking) at baseline 0.1%**

A 75 year old male living in Portugal

No lifetime alcohol problem or treatment

No panic disorder at baseline

AUDIT score of 0 at baseline

**Risk score 1%**

A 42 year old female living in Spain

No lifetime alcohol problem or treatment

No panic disorder at baseline

AUDIT score of 0 at baseline

**Risk score 5%**

A 36 year old male living in the Portugal

No lifetime alcohol problem or treatment

Panic disorder at baseline

AUDIT score of 5 at baseline

**Risk score 10%**

A 47 year old female living in Estonia

Lifetime alcohol problem or treatment

No panic disorder at baseline

AUDIT score of 3 at baseline

**Risk score 25%**

A 64 year old male living in the Netherlands

Lifetime alcohol problem or treatment

No panic disorder at baseline

AUDIT score of 6 at baseline

**Risk score 30%**

A 50 year old female living in the UK

No lifetime alcohol problem or treatment

Panic disorder at baseline

AUDIT score of 4 at baseline
